# Computational models for predicting liver toxicity in the deep learning era

**DOI:** 10.3389/ftox.2023.1340860

**Published:** 2024-01-19

**Authors:** Fahad Mostafa, Minjun Chen

**Affiliations:** ^1^ Department of Mathematics and Statistics, Texas Tech University, Lubbock, TX, United States; ^2^ Division of Bioinformatics and Biostatistics, National Center for Toxicological Research, U.S. Food and Drug Administration, Jefferson, AR, United States

**Keywords:** drug-induced liver injury (DILI), machine learning, deep learning, drug safety, predictive model

## Abstract

Drug-induced liver injury (DILI) is a severe adverse reaction caused by drugs and may result in acute liver failure and even death. Many efforts have centered on mitigating risks associated with potential DILI in humans. Among these, quantitative structure-activity relationship (QSAR) was proven to be a valuable tool for early-stage hepatotoxicity screening. Its advantages include no requirement for physical substances and rapid delivery of results. Deep learning (DL) made rapid advancements recently and has been used for developing QSAR models. This review discusses the use of DL in predicting DILI, focusing on the development of QSAR models employing extensive chemical structure datasets alongside their corresponding DILI outcomes. We undertake a comprehensive evaluation of various DL methods, comparing with those of traditional machine learning (ML) approaches, and explore the strengths and limitations of DL techniques regarding their interpretability, scalability, and generalization. Overall, our review underscores the potential of DL methodologies to enhance DILI prediction and provides insights into future avenues for developing predictive models to mitigate DILI risk in humans.

## 1 Introduction

Drug-induced liver injury (DILI) is a substantial safety concern, with a reported potential for more than 1,000 drugs or supplements to induce liver damage (Alempijevic et al., 2017; Zhu et al., 2018). DILI presents a significant challenge for healthcare professionals, pharmaceutical developers, and regulatory authorities (George et al., 2018), and frequently results in the discontinuation of drug candidates during their development (Weber and Gerbes, 2022). It also is a primary reason for the withdrawal of over 50 medications from the market (Devarbhavi, 2012; Wu et al., 2022) and ranks as a leading cause of acute liver failure in both the United States and Europe (Andrade et al., 2019). Despite notable advancements in drug safety, there is a continuing need for innovative approaches and methodologies to identify drugs candidates in development with potential hepatotoxicity in humans, and for reliable biomarkers to facilitate the early detection of DILI (Chen et al., 2014).

The need to enhance safety assessments in drug development has driven new approaches for predicting toxicity. Conventional methods often lack the precision and efficiency required to mitigate the risks associated with liver toxicity. However, machine learning, (ML), which includes Quantitative Structure-Activity Relationship (QSAR) modeling (Idakwo et al., 2019; Shin et al., 2023) as a pivotal component, harnesses extensive datasets, chemical structures, and biological assays to establish quantitative associations between molecular properties and toxicity outcomes (Wu et al., 2017). This approach has the potential to facilitate the detection of liver toxicity during the early stage drug development process, enabling screening of drug candidates and their analogs prior to chemical synthesis.

An advanced ML technique, deep learning (DL), signifies a transformative approach in the field of liver toxicity prediction, offering the potential for exceptionally accurate, data-driven insights. DL harnesses neural networks and extensive datasets, which encompass chemical data, biological assays, and omics information, to construct predictive models of outstanding performance (Xu et al., 2015; Goh et al., 2018). Its integration into liver toxicity prediction empowers researchers and pharmaceutical companies to identify potential risks associated with drug candidates at an early stage in the development process. Moreover, its capacity to analyze diverse and intricate data sources facilitates a better understanding of toxicity mechanisms. Consequently, DL not only advances patient safety by aiding in identifying harmful compounds, but also is cost-effective and contributes to the accelerated development of safer and more effective medications.

In this review, we focus on cutting-edge research using ML/DL applications to predict liver toxicity. We first examine the application of ML in liver toxicity prediction, with a particular emphasis on the development of QSAR models. Next, we provide a systematic evaluation of DL methods and their application for predicting liver toxicity, drawing comparisons with traditional ML approaches. Finally, we discuss the strengths and limitations of DL methods in interpretability, scalability, and generalization.

## 2 Machine learning for predicting liver toxicity

Machine learning (ML) algorithms have extensive applications in classification tasks, including the prediction of liver toxicity (Supplementary Table S1). In binary classification, compounds are typically categorized into two classes: a toxic class (commonly labeled class 1) and a non-toxic class (class 0). ML algorithms can learn from historical data and categorize new instances into one of these two classes by considering their observed characteristics, such as chemical structures. Among the various ML methods available, Naive Bayes Classifier (NBC), Support Vector Machines (SVM), and Random Forests are widely employed in this context.

### 2.1 Naive Bayes classifier

The Naive Bayes classifier (NBC) is a probabilistic ML algorithm widely used for both binary and multiclass classification tasks (Rish, 2001; Hastie et al., 2009). It is rooted in Bayes’ theorem, which quantifies the probability of an event based on prior knowledge of related events. The “Naive” component of its name comes from the assumption that input features are conditionally independent, simplifying calculations and enhancing computational efficiency. Thus, the NBC computes the conditional probability of a given instance belonging to a specific class by making the “naive” assumption of feature independence. Mathematically, it leverages Bayes’ theorem:

$$P\left(C_{k}|x\right)=\frac{P\left(C_{k}|x\right)\prod_{i=1}^{n}P\left({x_{i}|C}_{k}\right)}{P\left(x\right)},$$

where $C_{k}$ is the class, x is the feature vectors.

The NBC makes the “naive” assumption that features are independent given the class. This strong assumption might not hold in all real-world scenarios. However, despite this simplification, it often performs surprisingly well and is computationally efficient. Critical steps to train and use NBC are listed below:

1. Calculate Class Priors: Estimate the prior probabilities $P\left(C_{k}|x\right)$ for each class based on the training data.
2. Calculate Feature Probabilities: Estimate the conditional probabilities $P\left(x_{i}|C_{k}\right)$ for each feature and class pair based on the training data. This involves counting occurrences of features in each class.
3. Classification: Given a new instance with features $x_{1}{,x}_{2},\ldots {,x}_{n}$ , calculate the posterior probability for each class using Bayes’ theorem. The class with the highest probability is the predicted class.

Variations of NBCs are based on types of data and assumptions. Some common variations are:

• Gaussian Naive Bayes: Assumes features follow a Gaussian (normal) distribution.
• Multinomial Naive Bayes: Suited for discrete features like text data, and often used for document classification.
• Bernoulli Naive Bayes: Designed for binary feature data (presence/absence), and often used for text classification tasks.

NBC is a straightforward yet effective classifier, particularly suitable for binary classification tasks, provided that the assumption of feature independence is reasonably met. While it may not be the optimal choice for all data types, it serves as a standardized baseline classifier and is extensively employed in the prediction DILI through QSAR modeling (Ai et al., 2019; Williams et al., 2019; Wu Y. et al., 2021). For instance, Zhang et al. (2016) employed NBC to construct a computational model for assessing DILI risk. Their model exhibited a 94.0% accuracy in 5-fold cross-validation during the training phase, with a concordance rate of 72.6% on an external test set. They identified key molecular characteristics associated with DILI risk.

Tang et al. (2020) developed QSAR models for mitochondrial toxicity using five machine learning methods, including NBC along with various chemical signatures. They adopted a threshold moving strategy to rectify data imbalance and implemented consensus models to enhance prediction performance, achieving up to 88.3% accuracy in external validation. Notably, the study highlighted the significance of substructures such as phenol, carboxylic acid, nitro compounds, and aryl chloride in classification. In another work, Rao et al. (2023), proposed an integrated artificial intelligence (AI)/ML model that employed physicochemical properties and *in silico* off-target interactions to predict the severity of DILI for small molecules. They utilized data from 603 compounds categorized by the U.S. Food and Drug Administration (FDA) as Most DILI, Less DILI, and No DILI, and combined the NBC with other ML approaches to enhance DILI prediction, surpassing the performance of QSAR models based solely on chemical properties.

### 2.2 Support vector machine classifier

The Support Vector Machine (SVM) is a potent and versatile machine learning algorithm used for a range of tasks, including regression, binary, and multiclass classification, as is the case in predicting DILI through QSAR modeling with chemical structures (Li et al., 2020a; Tang et al., 2020; Wu Z et al., 2021; Rao et al., 2023). Its primary goal is to identify a hyperplane that maximizes the margin between the nearest data points from the two classes. The fundamental concept is to optimize this margin between classes, resulting in improved generalization to new, unseen data. These closest data points are referred to as “support vectors.” The margin is defined as the distance between the hyperplane and these support vectors. Mathematically, the SVM tries to solve the following optimization problem:

$$\underset{w,b}{\frac{1}{2}\min}{\left\|w\right\|}^{2}$$

Subject to

$$y_{i}\left(w∙x_{i}+b\right)\geq 1fori=1:n,$$

where:

• w is the weight vector perpendicular to the hyperplane.
• b is the bias term.
• $x_{i}$ are the feature vectors.
• $y_{i}$ are the class labels (1 or 0) for each data point.
• n is the number of data points.

The above optimization problem ensures that data points are correctly classified with a margin. Support vectors are the data points that lie on the margins or violate the margin constraint. In many cases, the data may not be linearly separable in the original feature space. To handle these cases, SVMs often use a kernel trick. A kernel function transforms the original feature space into a higher-dimensional space, where the data might become separable. Common kernel functions include:

• Linear Kernel: $K\left(x,x^{\prime }\right)=x∙x^{\prime }.$

• Polynomial Kernel: $K\left(x,x^{\prime }\right)={\left(x∙x^{\prime }+c\right)}^{d}.$

• Radial Basis Function (RBF) Kernel: $K\left(x,x^{\prime }\right)=e^{-\gamma {\left\|x-x^{\prime }\right\|}^{2}}.$

In some cases, the data might not be perfectly separable, or there could be outliers. In such situations, SVM employed a soft margin to allow for some misclassification by introducing a slack variable. The optimization problem becomes:

$$\underset{w,b}{\frac{1}{2}\min}{\left\|w\right\|}^{2}+C\sum_{j=1}^{n}\tau_{j}$$

Subject to:

$$y_{i}\left(w∙x_{i}+b\right)\geq 1-\tau_{j}fori,j=1:n,\tau_{j}\geq 0,$$

where C is a hyperparameter that controls the trade-off between maximizing the margin and minimizing misclassification. Major steps to train and apply the SVM Classifier are listed below.

1. Data Preparation: Gather and preprocess the data, ensuring it is properly labeled, and features are appropriately represented.
2. Choose a Kernel: Decide on a kernel function based on the data characteristics, which can be critical to improving accuracy in DILI predictions (Wu Y. et al., 2021).
3. Train the SVM: Use an optimization algorithm to find the optimal hyperplane parameters (weights w and bias b) that minimize the objective function.
4. Classification: Given a new instance with features x, calculate the decision function $f\left(x\right)=w∙x+b$ . If $f\left(x\right)>0$ , classify as class 1; if $f\left(x\right)<0$ , classify as class 0. SVMs can be computationally intensive for large datasets, and tuning the hyperparameters, such as the choice of kernel and the regularization parameter C, is essential for optimal performance.

SVM has been applied extensively in predicting DILI, particularly in scenarios with limited data, owing to its robust prediction accuracy and computational efficiency. Notable studies (Wang et al., 2019; Li et al., 2020b; Hemmerich et al., 2020; Mora et al., 2020; Wu Z. et al., 2021) have employed SVM for DILI prediction. In a comprehensive analysis conducted by Wu Y. et al. (2021), involving 14 sets of QSAR data and 16 ML algorithms, the radial basis function SVM (rbf-SVM) emerged as the top-performing method among all ML techniques, underscoring its efficacy in this domain.

However, certain limitations were associated with the SVM algorithm. SVM tends to be computationally expensive and may not be well-suited for very large datasets. When the dataset exhibits extra noise, such as overlapping target classes, SVM’s performance can be compromised. Furthermore, SVM may perform suboptimally when the number of features for each data point exceeds the number of training data samples. These considerations are critical when deciding on the suitability of SVM for specific DILI prediction tasks.

### 2.3 Random forest classifier

The Random Forest classifier is a robust ensemble ML algorithm frequently employed for both binary and multiclass classification tasks. It is an extension of the decision tree algorithm, having the primary objective of enhancing generalization and mitigating overfitting by forming an ensemble of multiple decision trees. In binary classification, the Random Forest classifier is designed to classify new instances into one of two classes based on their features. The process involves the construction of multiple decision trees during the training phase, and their collective predictions are amalgamated to reach the final classification decision. Key steps for training and applying the Random Forest classifier are outlined below.

1. Bootstrapped Sampling: For each tree in the forest, a random subset of the training data is selected with replacement. This process is known as bootstrapped sampling. It creates diversity among the trees, as each tree is trained on a slightly different data subset.
2. Random Feature Selection: At each split point in a decision tree, only a subset of the available features is considered for splitting. This introduces further randomness and prevents individual trees from relying on any one feature.
3. Tree Building: Each decision tree is constructed using the bootstrapped training data and random feature selection. The tree is grown until a stopping criterion is met, usually involving the maximum depth of the tree or the minimum number of samples required to split a node.
4. Voting for Classification: During prediction, each tree in the forest independently classifies the input data. The final classification decision is made by taking a majority vote among the individual tree predictions. In the case of binary classification, the class with the most votes wins.

Compared with other machine learning methods, random forest has several unique characteristics:

• Reduced Overfitting: The ensemble of trees helps to mitigate overfitting by averaging out the noise and biases present in individual trees.
• Improved Generalization: Random Forests are robust to outliers and noisy data due to the aggregation of multiple trees.
• Feature Importance: Random Forests can provide insights into feature importance by analyzing how much each feature contributes to the model’s performance.
• Non-linearity Handling: Random Forests can capture complex relationships in the data without requiring explicit feature extraction/selection.

Random Forest emerges as an invaluable machine learning technique for the classification of liver toxicity. Gadaleta et al. (2018) employed Random Forest classifiers and DRAGON molecular descriptors to create QSAR models designed to predict molecular initiating events leading to hepatic steatosis. They effectively used a Balanced Random Forest classifier, alongside the strategy of under-sampling, to construct robust QSAR models from unbalanced DILI datasets. Both techniques yielded comparable predictive results, achieving approximately 75% accuracy in toxicity prediction.

## 3 Deep learning for predicting liver toxicity

Deep learning (DL) represents a new class of machine learning methods characterized by the use of highly complex neural networks. Networks are structured in deeply nested architectures, often incorporating advanced operations like convolutions and multiple activation functions. These distinctive features empower DL with the unique capability to process raw input data and autonomously uncover hidden patterns for learning tasks. In the context of predicting liver toxicity, several DL methods are commonly employed for classification tasks. These methods deploy neural networks with diverse architectures and techniques to achieve precise and efficient classification (Supplementary Table S1). We provide a brief overview of various DL methods, including multi-layer perceptron (MLP), deep neural network (DNN), convolutional neural network (CNN), graph neural network (GNN), recurrent neural network (RNN), generative adversarial network (GAN), and transformer.

### 3.1 Multi-layer perceptron

The Multilayer Perceptron (MLP), also known as an Artificial Neural Network (ANN), is a fundamental neural network architecture used for a wide range of machine learning applications, including classification and regression. An MLP consists of multiple layers of artificial neurons, typically structured into an input layer, one or more hidden layers, and an output layer. Each neuron within a layer is connected to each neuron in the layers above and below it, creating a densely interconnected network. Connections between neurons, represented as weights (often denoted as W), are learned during the training process. The output of each neuron is determined by applying an activation function, such as the sigmoid, ReLU, or tanh function, to a weighted sum of its inputs. Mathematically, the output (O) of a neuron in a hidden or output layer is computed as follows:

$$y=f\left(\sum_{i=1}^{n}w_{i}.x_{i}+b\right),$$

where y is the output of the neuron. f is the activation function. $w_{i}$ represents the weight associated with the i-th input connection. $x_{i}$ is the i-th input to the neuron, and b is the bias term. The training process involves adjusting these weights and biases using techniques like backpropagation and gradient descent to minimize a loss function, allowing the MLP to learn complex relationships within the data.

MLPs are versatile and can approximate a wide range of functions, making them a popular choice for various ML applications. Cruz-Monteagudo et al. (2008) investigated computational approaches for predicting idiosyncratic hepatotoxicity using 3D chemical structures such as linear discriminant analysis (LDA) and ANNs. The RBF architecture was used in a neural network classification method that used the same descriptors as those in the LDA model. In the training series, this RBF neural network outperforms the LDA model, achieving an accuracy of 91.07%, sensitivity of 92.00%, and specificity of 90.32%. Examination of the Receiver Operating Characteristic (ROC) curve proved its continuously superior performance.

### 3.2 Deep neural networks

A Deep Neural Network (DNN) can be mathematically represented as a composition of functions (Schmidhuber, 2015). Given an input vector x , the output y of a DNN with L layers can be expressed as:

$$y=f_{L}\circ f_{L-1}\circ \ldots \circ f_{2}\circ f_{1}\left(x\right)$$

Each layer l applies a linear transformation $z_{l}=W_{l}a_{\left\{l-1\right\}}+b_{l}$ followed by an activation function $a_{l}=\sigma \left(z_{l}\right),$ where $W_{l}$ is the weight matrix and $b_{l}$ is the bias vector for that layer. The final output is obtained by applying an appropriate activation function at the last layer. Like MLP, DNN can be trained by adjusting the weights and biases to minimize a chosen loss function through techniques like backpropagation and optimization algorithms. DNNs are excellent for automatically extracting key features from large inputs, making them the perfect choice for transcriptomic data containing a wide variety of features.

DNNs (Hinton et al., 2006) have been effectively used to address the challenge of predicting various types of chemically induced liver injuries, including biliary hyperplasia, fibrosis, and necrosis, using DNA microarray data (Feng et al., 2019). Wang et al. (2019) used multi-task DNNs to evaluate gene and pathway-level feature selection strategies for these liver injuries. The DNN models exhibited high predictive accuracy and endpoint specificity, surpassing the performance of Random Forest and SVM models. In another study, Li et al. (2020b), developed a DNN model with eight layers using transcriptome profiles of human cell lines to predict DILI. The model leveraged a substantial binary DILI annotation dataset, achieving AUCs of 0.802 and 0.798 for the training and independent validation sets, respectively. These results outperformed traditional machine learning algorithms, including K-nearest neighbors, SVM, and Random Forest.

In a study conducted by Kang and Kang (2021), a DNN-based model was designed to predict DILI risk. This model used extended connectivity fingerprinting of diameter 4 (ECFP4) to represent molecular substructures. The data for this predictive model was meticulously collected from various sources, including publications like DILIrank and LiverTox. A model was developed through stratified 10-fold cross-validation, and the best DNN model showed an accuracy of 0.731, a sensitivity of 0.714, and a specificity of 0.750 when validated in the complete applicability domain. Jain et al. (2021) used a large-scale acute toxicity dataset encompassing over 80,000 compounds measured against 59 toxicity endpoints. They compared multiple single and multitask models using RF, DNN, CNN, and GNN approaches and found that multitask DL methods performed best.

### 3.3 Convolutional neural networks

Convolutional Neural Networks (CNNs) are mainly applied in image and speech recognition. These networks are well-suited for capturing spatial hierarchies and local patterns within images. CNNs typically incorporate convolutional layers for feature extraction, pooling layers for dimensionality reduction, and fully connected layers for classification. Architectures like AlexNet, VGG, ResNet, and InceptionNet have consistently demonstrated exceptional performance on various image classification tasks (Krizhevsky et al., 2012; Szegedy et al., 2015; Yamashita et al., 2018; Jain et al., 2021). Mathematically, CNN can be written as follows: Let X be the dataset having m number of images. The input feature size is denoted as n×n . Then the convolution layer is written as:

$$A_{ij}^{\left(l\right)}=\sigma \left(\sum_{p=1}^{f}\sum_{q=1}^{f}W_{p,q}^{\left(l\right)}A_{i+p-1,j+q-1}^{\left(l-1\right)}+b^{l}\right)$$

$$A^{\left(l\right)}=conv\left(A^{\left(l-1\right)},F^{\left(l\right)}\right)$$

Here, l=1,…,L are the number of convolution layers. Next, the CNN has the pooling layer:

$$P_{ij}^{\left(l\right)}=\max\;\left(A_{pi,pj,}^{\left(l\right)}A_{pi,pj+1}^{\left(l\right)},\ldots ,A_{pi+p-1,pj+p-1}^{\left(l\right)}\right)$$

$$P^{\left(l\right)}=maxpool\left(A^{\left(l\right)},P\right)$$

Flatten the pooled feature maps to obtain a vector of size F . Now the fully connected layer is defined as:

$$Z^{\left(l\right)}=W^{\left(l\right)}{∙A}^{\left(l-1\right)}+b^{\left(l\right)}$$

$$Z^{\left(l\right)}=FC\left(A^{\left(l-1\right)},H^{\left(l\right)}\right)$$

The output layer has a single neuron for binary classification or multiple neurons for multi-class classification:

$$Y=\sigma \left(Z^{\left(L\right)}\right)$$

A suitable loss function, such as binary cross-entropy, is used for classification. The network is trained using gradient descent-based optimization to minimize the chosen loss function.

CNN was also used for DILI prediction (Nguyen-Vo et al., 2020; Chen X. et al., 2022). Nguyen-Vo et al. (2020) introduced a novel computational model for the prediction of DILI utilizing CNNs and molecular fingerprints based on 1,597 compounds. The model came up with an average accuracy of 0.89, a Matthews correlation coefficient of 0.80, and an AUC of 0.96.

### 3.4 Graph Neural Networks

Graph Neural Networks (GNNs) are a class of neural networks explicitly tailored for operating on graph data structures. They are particularly well-suited for tasks involving graphs, such as social network analysis, chemical structure analysis, and computational vision (Zhou et al., 2020). Node-level tasks are used in DILI prediction and chemical structure analysis, and involve predicting the properties or characteristics of individual chemical components, such as molecules, within a graph or network structure.

To learn node representations, GNNs combine information from nearby nodes, effectively capturing intricate relationships in graphs. For example, let $G=\left(V,E\right)$ be the molecular graph, where V is the set of nodes (atoms) and E is the set of edges (bonds). The graph convolutional layer updates node representations based on their neighbors’ features. Let X be the initial node features (molecular fingerprint-embedded features) for all nodes in the graph. The output of the l− th graph convolutional layer can be represented as $X^{\left(l\right)}$ using the following equation:

$$X^{\left(l\right)}=\sigma \left({\hat{D}}^{-\frac{1}{2}}\hat{A}{\hat{D}}^{-\frac{1}{2}}X^{\left(l\right)}W^{\left(l\right)}\right),$$

Here, $\hat{A}=A+I$ is the adjacency matrix of the graph with added self-loops, $\hat{D}$ is the diagonal of matrix $\hat{A},$ $W^{\left(l\right)}$ is the learnable weights for l− th layers, and σ is the activation function. Pooling or aggregation layers were incorporated to combine node features across different neighborhoods. Similar with the CNN architecture, one or more fully-connected layers were used to learn higher-level representations from the aggregated features.

The final layer produces the network’s output. Depending on the task (e.g., regression, classification), the number of neurons and the activation function in the output layer can be adjusted. The forward pass through the GNN can be represented mathematically as written below. Let $X^{\left(0\right)}$ be the molecular fingerprint-embedded features. The graph convolution is:

$$\begin{array}{c} X^{\left(l\right)}=\sigma \left({\hat{D}}^{-\frac{1}{2}}\hat{A}{\hat{D}}^{-\frac{1}{2}}X^{\left(l\right)}W^{\left(l\right)}\right)\\ \text{Aggregated Features}=\text{Pooling}/\text{Aggregation }\left(X^{\left(l\right)}\right)\\ D_{1}=\text{Dense }\left(\text{Aggregated Features},\text{neurons},\text{activation}\right),\ldots ,\\ D_{k}=\text{Dense }\left(D_{k-1},\text{neurons},\text{activation}\right)\\ \text{Output}=\text{Dense }\left(D_{k},\text{output neurons},\text{output activation}\right) \end{array}$$

Here, k represents the number of dense layers in the network.

GNNs have demonstrated their efficacy in addressing node-level tasks related to DILI predictions (Hwang et al., 2020). Ma et al. (2020) used a MV-GNN based model as a backbone to propose a property augmentation approach to involving more data for four datasets with liver toxicity-relevant properties. The GNN-based approach significantly outperformed existing baselines on DILI datasets, achieving an impressive 81.4% accuracy using cross-validation with random splitting. Lim et al. (2023) introduced a novel technique known as supervised subgraph mining (SSM). SSM effectively identifies explicit subgraph features through iterative optimization of graph transitions. This approach surpasses conventional machine learning methods such as SVM, Random Forest, k-Nearest Neighbors, and deep learning neural networks in DILI classification using two datasets, DILIst and TDC-benchmark. By employing structure-based pattern matching, the proposed approach can also identify subgraph characteristics associated with specific medication groups.

### 3.5 Recurrent neural networks

Mathematically, a recurrent neural network (RNN) can be represented as follows: at each time step t , the RNN takes an input vector $x_{t}$ , and computes the hidden state $h_{t}$ and the output $y_{t}$ using the following equations:

$$h_{t}=f\left(W_{hh}*h_{t-1}+W_{hx}*x_{t}+b_{h}\right)$$

$$y_{t}=g\left(W_{yh}*h_{t}+b_{y}\right)$$

Here, $h_{t}$ represents the hidden state at time t , $x_{t}$ is the input at time t , $y_{t}$ is the output at time t , $W_{hh}$ , $W_{hx}$ , $W_{yh}$ are weight matrices, $b_{h}$ and $b_{y}$ are bias vectors, f and g are activation functions (typically sigmoid or hyperbolic tangent for f and softmax for g ). The hidden state $h_{t}$ captures information from previous time steps, allowing RNNs to model temporal dependencies in sequential data.

Xu et al. (2015) employed undirected graph recursive neural networks (UGRNN) to develop DL models for predicting DILI for drugs and small moleculars. Their DL-combined model outperformed ANN and DNN models, achieving an accuracy of 86.9% and an AUC of 0.955 when predicting the DILI of 198 drugs in the external validation set. The model also successfully identified important molecular substructures relevant to DILI, demonstrating the power of DL in this context. In another study Ruiz Puentes et al. (2021), investigators proposed using PharmaNet, a machine learning method that employs RNNs, to search for novel pharmaceutical candidates. PharmaNet was applied to discover ligands for 102 cell receptors and achieved impressive performance with a 97.7% Receiver Operating Characteristic curve-Area Under the Curve (ROC-AUC).

### 3.6 Generative adversarial network

Generative Adversarial Network (GAN) is an advanced generative model composed of two neural networks: a generator and a discriminator. These networks are trained in opposition to each other. The generator’s objective is to create synthetic data that is virtually indistinguishable from genuine data, while the discriminator’s role is to differentiate between real and generated data. GANs operate through a minimax game where the generator and discriminator compete. As training progresses, the generator becomes increasingly skilled at generating realistic data, while the discriminator becomes better at distinguishing between real and fake data. This dynamic process drives the generator to produce high-quality synthetic data, establishing GANs as a foundational technology in a wide range of applications, such as picture production, style transfer, and data augmentation.

Chen Z. et al. (2022) developed Tox-GAN, which employed deep GANs to generate fresh animal study results without the need for extra tests. They demonstrated its effectiveness by creating transcriptome profiles with remarkable similarity (0.997 ± 0.002 in intensity and 0.740 ± 0.082 in fold change) to real-world data obtained from rat liver toxicogenomic studies. In a related study, Li et al. (2023) introduced the TransOrGAN framework, which aims to map gene expression patterns across multiple rodent organs, sexes, and ages. TransOrGAN generated synthetic transcriptomic profiles with an average cosine similarity of 0.984 compared to their corresponding real profiles. This proof-of-concept study involved 288 samples from nine different organs, showcasing the potential of TransOrGAN to generate realistic transcriptomic data for various research applications.

### 3.7 Transformers

The field of Natural Language Processing (NLP) has undergone a transformative shift with the introduction of transformer-based models (Kang et al., 2020). These models have enabled the automatic analysis and comprehension of text data in scientific literature. In the domain of DILI studies, NLP models have proven to be valuable tools for extracting insights from textual sources.

Zhan et al. (2022) developed NLP techniques specifically for biomedical texts, allowing the automated processing of 28,000 titles and abstracts retrieved from the PubMed database. By comparing five different text embedding techniques, they found that the model using term frequency-inverse document frequency and logistic regression performed best, with an accuracy of 0.957 on the validation set. Wu Z. et al. (2021) employed a NLP approach based on Bidirectional Encoder Representations from Transformers (BERT) to classify DILI and decipher the meanings of complex text in drug labeling documents. This AI-based model utilized BERT’s power to enhance understanding of text data, particularly in the context of drug safety assessments.

## 4 Comparison of machine learning and deep learning for DILI prediction

**FIGURE 1:**
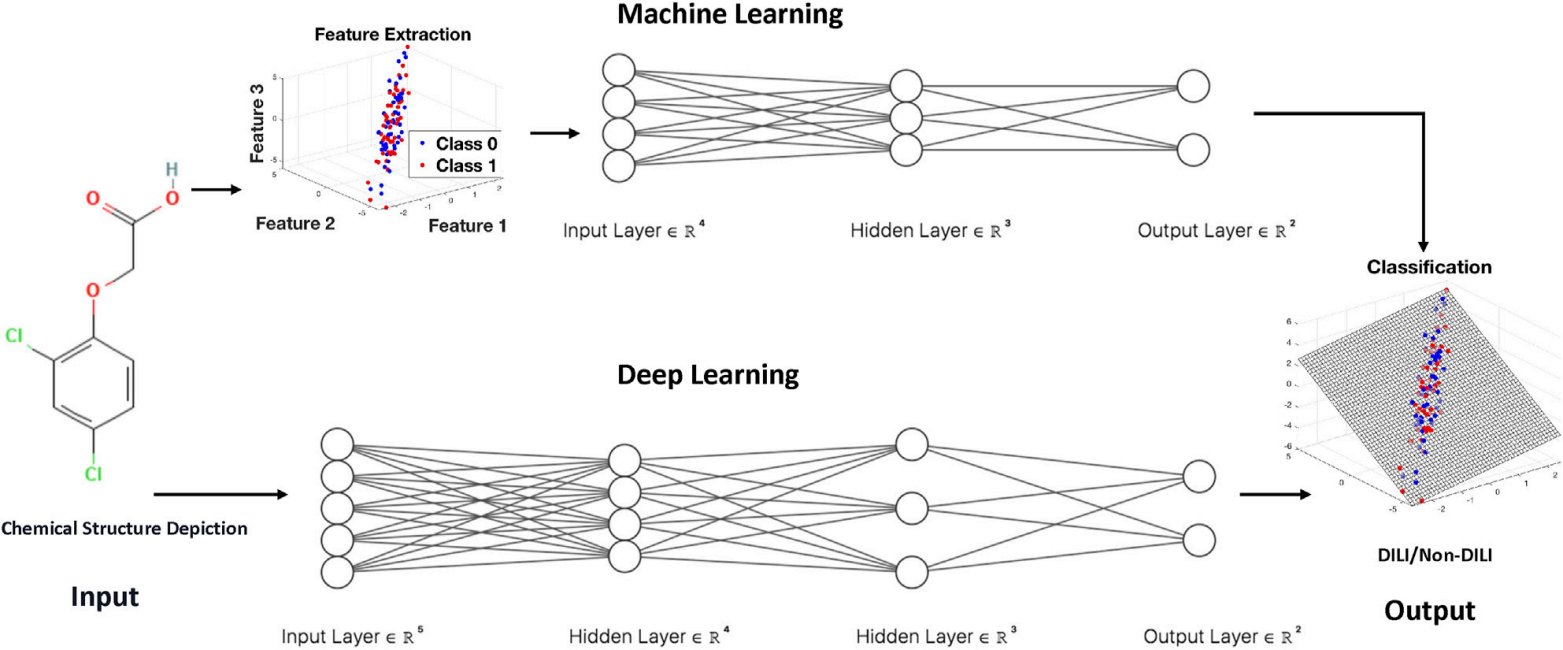
The general flowcharts for machine learning and deep learning techniques for developing models to predict DILI.

Machine learning and deep learning techniques have emerged as powerful tools for developing models to predict DILI (Table 1). Machine learning uses algorithms to discover patterns and make predictions based on labeled data, whereas deep learning, a subset of machine learning, uses artificial neural networks to replicate the sophisticated functioning of the human brain. Machine learning algorithms analyze a set of predefined features to identify patterns associated with liver injury in the context of DILI prediction, whereas deep learning models can automatically extract intricate features from raw data, providing a more nuanced understanding of complex relationships. The major distinction between the two is in the level of abstraction and data representation (Figure 1). Machine learning is based on feature engineering, in which the algorithm needs to select important features from high-dimensional dataset, whereas deep learning can develop hierarchical representations from raw data, possibly catching subtle nuances that typical machine learning algorithms may overlook. Both approaches provide important contributions to improving our ability to detect and alleviate DILI, giving essential insights for drug development and patient safety.

**TABLE 1:** Comparative analysis of machine learning and deep learning for DILI prediction.

	Machine learning	Deep learning
Definition	Machine learning, as an application and subset of artificial intelligence, enables systems to autonomously learn from experiences and improve without manual intervention. Machine learning primarily generates outputs in the form of numerical values, such as score classifications	In contrast, deep learning is essentially a subset of machine learning that intricately connects recurrent neural networks and artificial neural networks. Deep learning produces outputs ranging from free-form elements, such as unrestricted sound and text, to numerical values
Data uses and presentation	Machine learning utilizes unstructured data and information, resulting in distinct data representation scenarios. It involves handling thousands of diverse data points, contributing to its learning process	Deep learning, leveraging artificial neural networks, introduces a different data representation paradigm, emphasizing neural networks. It is characterized by a vast amount of data, incorporates millions of data points, facilitating a more nuanced understanding of patterns and relationships. Deep learning models, especially deep neural networks, often require large amounts of labeled data for training
Algorithm	Machine learning employs a variety of automated algorithms, transforming them into numerous model functions capable of predicting future actions based on data patterns. Feature extraction is important for ML algorithms. Traditional machine learning models often have lower computational requirements compared to deep learning models	In contrast, deep learning relies on neural networks to transport input through multiple processing levels, elucidating the characteristics and relationships within the current dataset. However, it is not necessary to extract or select important features for deep learning algorithms because it can be adjusted by weights in the hidden layers of the network
Application of DILI prediction	Machine learning stays competitive on identifying hidden patterns from a small amount of input dataset. It assists in various aspects of DILI prediction and management	Deep learning excels in resolving complex machine learning challenges within a system, and its efficacy for DILI prediction will become more prominent with the progress of data accumulation in the field

## 5 Conclusion

Deep learning approaches have indeed shown significant promise in predicting DILI, leveraging the advantages of large datasets and the ability to capture intricate patterns. In the context of QSAR modeling, DL methods have often been reported to outperform conventional machine learning methods. However, it is essential to recognize that DL’s superiority is not always guaranteed and can depend on the specific characteristics of the dataset and the problem. For instance, Liu et al. (2018) pointed out that global performance metrics, which typically show DNNs as superior to conventional machine learning, may not be appropriate for datasets with highly imbalanced sample distributions. They argued that for highly toxic chemicals, DNNs trained on all samples often perform worse than indicated by global performance metrics.

Imbalanced datasets can lead to misrepresentations of the actual performance, especially in cases where the minority class (highly toxic chemicals, in this example) is of particular interest. Similarly, Russo et al. (2018) compared DNN with conventional machine learning algorithms, including Naive Bayes, AdaBoost Decision Tree, Random Forest, and SVM, in the development of QSAR models for predicting endocrine disrupting endpoints using up to 7,500 compounds. Their results revealed that while DNNs may achieve higher accuracy on the training set, they did not consistently outperform classic machine learning methods in 5-fold cross-validation and predictions on external test sets. The performance of machine learning models can be influenced by various factors, including the nature of the data, the choice of molecular descriptors, and the specific problem being addressed.

Deep learning has specific characteristics for toxicity prediction. Scalability is a primary one, since DL models can handle vast amounts of data and understand nuanced correlations, enabling the discovery of small DILI risk variables that older approaches may overlook. Furthermore, by collecting latent characteristics across varied datasets, these models can accomplish impressive generalization, boosting the capacity to predict DILI across different chemicals and patient groups. However, interpretability is a key weakness of DL in this scenario. Because the models are intrinsically complex, deciphering the precise biological or chemical elements leading to DILI forecasts is difficult, limiting one’s capacity to grasp the underlying processes. Additionally, DL also requires a large amount of high-quality data for training, and like machine learning, is also prone to overfitting when the training data is noisy or when the model is too complex.

Researchers and practitioners in this field must carefully consider these advantages and challenges when choosing and implementing DL approaches for toxicity prediction. Balancing the needs for accuracy and interpretability is crucial in improving our understanding and prediction of DILI and other toxicities.
