# Supplementary material for: Computational models for predicting liver toxicity in the deep learning era
Source: Front Toxicol. 2024 Jan 19;5:1340860. doi: 10.3389/ftox.2023.1340860 (PMC10834666; doi:10.3389/ftox.2023.1340860)
Supplement: Supplementary file 1 [file Table1.docx]

Supplemental table 1. Selected models by machine learning/deep learning to predict liver toxicity

| **References** | **Machine learning/**  **Deep learning** | **Endpoints for predicting** | **Predictors for modeling** | **Samples for modeling** | **Overall performance** |
| --- | --- | --- | --- | --- | --- |
| Zhang, Hui, et al. (2016) ^1^ | NBC | DILI potential in humans | Molecular descriptors | 420 and 84 drugs for training and test | 94% accuracy in CV; 72% accuracy in test |
| Hong, Huixiao, et al. (2017) ^2^ | DF | DILI potential in humans | Molecular descriptors | 721 drugs for training | 72.9% accuracy in CV |
| Williams, Dominic P., et al (2019)^3^ | NBC | DILI potential in humans | in vitro assays and physicochemical properties | 96 compounds for training | 86% balanced accuracy in CV |
| Hammann, Felix, et al. (2019) ^4^ | ANN | DILI potential in humans | Molecular descriptors | 576 drugs for training | 89% corrected classification rates in CV |
| Minerali, Eni, et al. (2020) ^5^ | NB | DILI potential in humans | Molecular descriptors | 938 drugs for training | 74.8% accuracy in CV |
| Cotterill, J., et al. (2020) ^6^ | LDA | Hepatic steatosis in humans and animals | Molecular descriptors | 207 compounds for training | 70% accuracy in CV |
| Tang, Weihao et al. (2020)^7^ | SVM, NBC, RF, LDA and consensus models | Mitochondrial toxicity | Molecular descriptors | 3849 and 962 for training and test | 88.3% accuracy in test |
| Jaganathan, Keerthana et al. (2021) ^8^ | SVM | DILI potential in humans | Molecular descriptors | 1253 and 208 for training and test | 81.1% accuracy in CV and 75.6% accuracy in test |
| Rao, Mohan, et al. (2023) ^9^ | SVM, RF, LDA, KNN, and NBC | DILI potential in humans | Molecular descriptors and physicochemical properties | 603 compounds for training | 0.88 AUC in CV |
| Xu, Youjun, et al. (2015) ^10^ | MLP | DILI potential in humans | Molecular descriptors | 475 and 198 drugs for training and test | 70.3% accuracy in test |
| Feng, Chunlai, et al. (2019) ^11^ | MLP and SVM | Liver toxicity in rats | Gene expression data | 790 and 198 samples for training and test | 97.1% accuracy in test |
| Wang Hao et al. (2019) ^12^ | DNN | Liver toxicity in rats | Gene expression data | about 2000 and 300 compounds for training and test | 74% accuracy in CV |
| Hwang, D., et al (2020) ^13^ | GNN | DILI potential in humans | Molecular descriptors and gene expression data | 151 drugs for training | 77.3% accuracy in CV |
| Chierici, Marco, et al. (2020) ^14^ | Deep learning networks | DILI potential in humans | Gene expression data | 180 and 86 drugs for training and test | 0.12 Matthews correlation coefficient in test |
| Ma H et al. (2020) ^15^ | GNN | DILI potential in humans | Graph of chemical structure | 479 drugs for training | 81.4% accuracy using in CV |
| Nguyen-Vo TH et al. (2020) ^16^ | CNN | DILI potential in humans | Molecular descriptors | 1597 and 322 compounds for training and test | 84% accuracy in test |
| Li, Ting, et al. (2020) ^17^ | KNN, SVM, RF, DNN | DILI potential in humans | Molecular descriptors | 753 and 249 drugs for training and test | 65.8% balanced accuracy in test |
| Chen Z et al.(2022) ^18^ | ResNet18 | DILI potential in humans | Molecular descriptors | 1299 and 147 drugs for training and test | 97.6% accuracy in test |
| Lim, Sangsoo, et al. (2023) ^19^ | GNN | DILI potential in humans | Graph of chemical structures | 748 and 249 drugs for training and test | 68.7% accuracy in CV |

Abbreviation: DF: Decision forest; NBC: Naıve Bayes classifier; KNN: K- nearest neighbor; LDA: linear discriminate analysis; SVM: support vector machine; ANN: artificial neural network; MLP: multi-layer perceptron; DNN: deep neural network; CNN: convolutional neural network; GNN: graph neural network; CV: cross-validation;

1 Zhang, H. *et al.* Predicting drug-induced liver injury in human with Naïve Bayes classifier approach. *Journal of computer-aided molecular design* **30**, 889-898 (2016).

2 Hong, H., Thakkar, S., Chen, M. & Tong, W. Development of decision forest models for prediction of drug-induced liver injury in humans using a large set of FDA-approved drugs. *Scientific reports* **7**, 17311 (2017).

3 Williams, D. P., Lazic, S. E., Foster, A. J., Semenova, E. & Morgan, P. Predicting drug-induced liver injury with Bayesian machine learning. *Chemical research in toxicology* **33**, 239-248 (2019).

4 Hammann, F., Schöning, V. & Drewe, J. Prediction of clinically relevant drug‐induced liver injury from structure using machine learning. *Journal of Applied Toxicology* **39**, 412-419 (2019).

5 Minerali, E., Foil, D. H., Zorn, K. M., Lane, T. R. & Ekins, S. Comparing machine learning algorithms for predicting drug-induced liver injury (DILI). *Molecular pharmaceutics* **17**, 2628-2637 (2020).

6 Cotterill, J., Price, N., Rorije, E. & Peijnenburg, A. Development of a QSAR model to predict hepatic steatosis using freely available machine learning tools. *Food and Chemical Toxicology* **142**, 111494 (2020).

7 Tang, W., Chen, J. & Hong, H. Discriminant models on mitochondrial toxicity improved by consensus modeling and resolving imbalance in training. *Chemosphere* **253**, 126768 (2020).

8 Jaganathan, K., Tayara, H. & Chong, K. T. Prediction of drug-induced liver toxicity using SVM and optimal descriptor sets. *International Journal of Molecular Sciences* **22**, 8073 (2021).

9 Rao, M. *et al.* AI/ML Models to Predict the Severity of Drug-Induced Liver Injury for Small Molecules. *Chemical Research in Toxicology* (2023).

10 Xu, Y. *et al.* Deep learning for drug-induced liver injury. *Journal of chemical information and modeling* **55**, 2085-2093 (2015).

11 Feng, C. *et al.* Gene expression data based deep learning model for accurate prediction of drug-induced liver injury in advance. *Journal of chemical information and modeling* **59**, 3240-3250 (2019).

12 Wang, H., Liu, R., Schyman, P. & Wallqvist, A. Deep neural network models for predicting chemically induced liver toxicity endpoints from transcriptomic responses. *Frontiers in pharmacology* **10**, 42 (2019).

13 Hwang, D., Jeon, M. & Kang, J. in *2020 IEEE International Conference on Big Data and Smart Computing (BigComp).* 323-329 (IEEE).

14 Chierici, M., Francescatto, M., Bussola, N., Jurman, G. & Furlanello, C. Predictability of drug-induced liver injury by machine learning. *Biology direct* **15**, 1-10 (2020).

15 Ma, H. *et al.* Deep graph learning with property augmentation for predicting drug-induced liver injury. *Chemical Research in Toxicology* **34**, 495-506 (2020).

16 Nguyen-Vo, T.-H. *et al.* Predicting drug-induced liver injury using convolutional neural network and molecular fingerprint-embedded features. *ACS omega* **5**, 25432-25439 (2020).

17 Li, T., Tong, W., Roberts, R., Liu, Z. & Thakkar, S. DeepDILI: deep learning-powered drug-induced liver injury prediction using model-level representation. *Chemical research in toxicology* **34**, 550-565 (2020).

18 Chen, Z. *et al.* ResNet18DNN: prediction approach of drug-induced liver injury by deep neural network with ResNet18. *Briefings in Bioinformatics* **23**, bbab503 (2022).

19 Lim, S. *et al.* Supervised chemical graph mining improves drug-induced liver injury prediction. *iScience* **26** (2023).
